# The interstitial compartment as a therapeutic target in heart failure

**DOI:** 10.3389/fcvm.2022.933384

**Published:** 2022-08-17

**Authors:** Doron Aronson

**Affiliations:** Department of Cardiology, Rambam Health Care Campus, B. Rappaport Faculty of Medicine, Technion Medical School, Haifa, Israel

**Keywords:** heart failure, congestion, interstitial, lymphatic system, Starling equation

## Abstract

Congestion is the single most important contributor to heart failure (HF) decompensation. Most of the excess volume in patients with HF resides in the interstitial compartment. Inadequate decongestion implies persistent interstitial congestion and is associated with worse outcomes. Therefore, effective interstitial decongestion represents an unmet need to improve quality of life and reduce clinical events. The key processes that underlie incomplete interstitial decongestion are often ignored. In this review, we provide a summary of the pathophysiology of the interstitial compartment in HF and the factors governing the movement of fluids between the interstitial and vascular compartments. Disruption of the extracellular matrix compaction occurs with edema, such that the interstitium becomes highly compliant, and large changes in volume marginally increase interstitial pressure and allow progressive capillary filtration into the interstitium. Augmentation of lymph flow is required to prevent interstitial edema, and the lymphatic system can increase fluid removal by at least 10-fold. In HF, lymphatic remodeling can become insufficient or maladaptive such that the capacity of the lymphatic system to remove fluid from the interstitium is exceeded. Increased central venous pressure at the site of the thoracic duct outlet also impairs lymphatic drainage. Owing to the kinetics of extracellular fluid, microvascular absorption tends to be transient (as determined by the revised Starling equation). Therefore, effective interstitial decongestion with adequate transcapillary plasma refill requires a substantial reduction in plasma volume and capillary pressure that are prolonged and sustained, which is not always achieved in clinical practice. The critical importance of the interstitium in the congestive state underscores the need to directly decongest the interstitial compartment without relying on the lowering of intracapillary pressure with diuretics. This unmet need may be addressed by novel device therapies in the near future.

## Introduction

The expansion of extracellular volume is central to the pathophysiology of heart failure (HF) and other edematous disorders, resulting in signs and symptoms (edema, dyspnea, orthopnea) commonly referred to as congestion (1–4). Edema is defined as excess free, mobile fluid in the interstitial space (5). In ambulatory patients with HF, edema and other signs of fluid overload are strongly related to patient-assessed quality of life and future adverse events (4). Increasing signs and symptoms of congestion are the main reasons why patients with acute heart failure seek urgent medical care (2).

Most patients who are hospitalized with worsening heart failure do not have a new, acute disorder. Rather, they present with decompensation of chronic underlying ventricular dysfunction as a consequence of progressive fluid retention and increase in cardiac filling pressures in the preceding weeks (6–8).

Congestion is therefore the single most important contributor to heart failure decompensation and the need for hospital admission (3, 9–11). Alleviation of dyspnea and congestion and reduction in readmissions for heart failure constitute a major treatment goal in acute HF and key measures of treatment efficacy in recent practice guidelines (12) and acute HF trials (13, 14). However, many patients have only partial relief of dyspnea and congestion, even with the implementation of guideline-recommended therapies (10, 15–17). Patients with acute HF and fluid overload with suboptimal response to diuretics are at risk for inadequate decongestion, which is associated with worse outcomes (4, 18–26). Consequently, decongestion in the outpatient setting and post-hospital discharge represents an unmet need to improve quality of life and reduced clinical events (3, 23, 27).

Beyond the classical paradigm of salt and fluid retention, another form of congestion can be triggered by fluid redistribution rather than by fluid accumulation *via* rapid changes in venous capacitance and/or by a rapid increase in systemic pressure and systemic vascular resistance and afterload mismatch (28, 29). Such patients present primarily with pulmonary edema and acute elevation of filling pressures, have minimal systemic edema, and are often systemically euvolemic.

The abdominal (splanchnic) veins are considerably more compliant than veins of the extremities or skin, and therefore, store 20–50% of the total blood volume (30). As such, splanchnic veins account for a much greater proportion of the total capacitance in the venous system and play an important role in intravascular volume shifts (31).

The splanchnic veins contain large numbers of α1 and α2 adrenergic receptors and are highly sensitive to sympathetic stimulation (29, 32). Sympathetic activation of the splanchnic capacitance veins results in a rapid translocation (within seconds) of the splanchnic venous reservoir into the central vascular compartment leading to an increase in effective circulatory volume (33). Such autonomically mediated volume shift can underlie the development of acute HF, which occurs without an increase in total blood volume (32). Importantly, there is an interaction between these two forms of congestion as sodium and water retention amplify the hemodynamic effects of vascular capacitance (32).

The current medical practice relies exclusively on diuretic therapy (enhancing renal removal of salt and water) to treat congestion. As stated in a 2019 position statement of the Heart Failure Association of the European Society of Cardiology, “Other than ultrafiltration, the only pathway to get rid of sodium and water is through increased renal natriuresis and diuresis” (34).

Previous attempts to enhance decongestion while preserving renal function using medical therapies, such as low-dose dopamine, nesiritide, rolofylline (an adenosine A1-receptor antagonist) (35, 36), or ultrafiltration (37), failed to improve clinical outcomes.

There is the recent emphasis on the optimal management of diuretic resistance (defined as an inadequate natriuretic response despite an adequate diuretic regimen), as a major driver of suboptimal decongestion (2, 27). The usual practice to overcome diuretic resistance remains the administration of increasing doses of loop diuretics or adding thiazide diuretic or aldosterone-receptor antagonists. This approach may lead to intravascular volume depletion, electrolyte abnormalities, and further worsening of renal function (see below) (38, 39). Attempts to effectively treat congestion in diuretic-resistant patients while preserving renal function are often met with limited clinical success and require therapeutic decisions that reflect a compromise between potential benefits and harms (19). Therefore, there remains an unmet need for renal adjuvant therapies that enhance the ability to attain adequate decongestion and preserve renal function (12, 40).

Normally, the fluid capacity of the interstitial compartment is ≈3-fold to 4-fold of the intravascular compartment (1). Skin and skeletal muscle contain most of the extracellular volume (41). In HF, the ratio of the interstitial compartment volume to plasma volume (PV) can increase by several folds (1). Consequently, the interstitial compartment accounts for most of the excess volume in congested HF patients (1) and most of the fluid loss with diuretic therapy (42, 43). Therefore, it is important to understand the mechanisms that govern fluid mobilization within the interstitial compartment. Recently, there is an increasing interest in device-based therapies targeting congestion (44), with specific devices targeting extravascular fluid overload.

## The interstitial volume–pressure relationship

The interstitial compartment is composed of several structural elements, collectively called the extracellular matrix (ECM) that consists of three major components (45): (1) the fibrous collagen network (e.g., types I and III collagen fibers); (2) the microfibril–elastin fibrous system (elastin, fibronectin); (3) ground substance formed by proteoglycans, including glycosaminoglycans (GAGs) and a fine meshwork of polymerized hyaluronic acid and other mucopolysaccharides.

The GAGs are linear polymers of disaccharides covalently bound to a protein backbone, thus forming large macromolecules which interact with the fibrous components of the ECM. GAGs are negatively charged and therefore capable of binding cations, particularly sodium ions.

The interstitial volume (*V*_*i*_) is determined by the interstitial pressure and the interstitial pressure–volume relationship (46). Connective tissue cells (fibroblasts) together with ECM fibers apply tensile forces such that the tissue is held under mild compression. The tensile forces are mediated by integrins, such as the β1-integrin and the collagen-binding integrin α2β1, and laminin. Tensile forces compact the ECM and, under normal conditions, resist transcapillary fluid flux into the interstitium (45, 47, 48).

By contrast, the polyanionic osmotically active glycosaminoglycans, particularly hyaluronan, attracts water and exerts imbibition pressure (akin to a sponge). This inherent tendency to expand (glycosaminoglycan swelling) promotes interstitial volume expansion and decreased interstitial fluid (ISF) pressure (47). Normally, this tendency to swell is restrained by the fiber networks in the tissues (45). The properties of its structural elements enable the ECM to regulate the interstitial hydrostatic pressure (*P*_*i*_) and to modulate the volume–pressure relationship of the interstitium (49).

**FIGURE 1:**
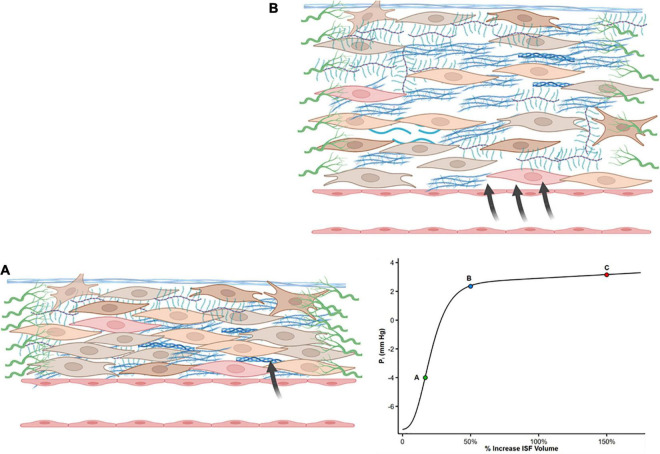
Shape of the interstitial volume–pressure relationship. Zero represents euvolemia. At low interstitial volume, compliance is low, and an increase in volume causes pressure to rise steeply (from **A** to **B**), opposing further filtration. This steep increase in pressure is produced by tensile forces applied by fibroblasts and extracellular matrix. With further interstitial fluid accumulation, typically interstitial volume 20–50% above euvolemia, interstitial compliance markedly increases as a result of structural changes, and large changes in volume result in a minimal increase in pressure (from Point B to C). Modified from references (50, 51, 55, 121).

The slope of the interstitial pressure–volume relationship (or compliance, defined as Vi/Pi) reflects the interaction between these two dynamic opposing forces (49, 50). *P*_*i*_ is slightly negative to zero (–4 to 0 mmHg) (51–53) particularly in subcutaneous tissue and lungs (54). Normally, a small increase in ISF content leads to steep increases in interstitial tensile stress (Figure 1A). Thus, a small increase in net filtration and *V*_*i*_ will be counteracted by a marked increase in *P*_*i*_ (51, 55), resisting further capillary filtration and edema. However, once interstitial volume rises by approximately 20–50%, or *P*_*i*_ increases to slightly positive values (52), the interstitium becomes highly compliant (Figure 1B). Large changes in volume now marginally increase *P*_*i*_ and allow progressive capillary filtration and edema (Figure 1, Point C on the curve).

Guyton who originally described the interstitial volume–pressure relationship recognized the importance of the *shape* of the curve and its clinical implication as it initially opposes edema formation (in concert with the lymphatic flow) but also allows the accumulation of large quantities of fluid in the interstitial compartment once ISF pressure has risen into the positive range (56).

The reason for the sudden increase in interstitial compliance is not entirely clear. Long-term edema may lead to relaxation of tissue elements or disrupt ECM compaction thus altering the integrity of the GAG network by reducing integrin receptor interactions with ECM (55, 56). Such ECM remodeling may result in a decreased tensile stress, and thus, a high compliance state of the interstitial matrix that no longer restrains edema formation. Whether long-standing edema is sufficient to induce fibrosis (hence increasing mechanical stress and stiffness of the ECM) remains controversial (57).

The interstitial volume–pressure relationship (Figure 1) has an important implication for lymphatic compensation in HF. At the negative pressure region of the interstitial pressure–volume curve, there is a direct relationship between lymph flow and ISF pressure (58). As interstitial compliance increases with tissue hydration (due to matrix remodeling), higher volume must be filtered to change *P*_*i*_, and this can dampen the driving forces leading to lower lymph flows relative to those obtained when *P*_*i*_ rapidly increases in the low compliance range (59). At a higher positive *P*_*i*_, lymph flow reaches a maximum value, and further elevation of interstitial volume produces no further augmentation (and even a reduction) of lymph flow (58–60).

## Starling forces

Classically, transcapillary filtration is determined by capillary pressure (*P*_*c*_) and interstitial protein osmotic pressure (*π_*i*_*), while a counteracting absorptive force is exerted by plasma protein osmotic pressure (*π_*p*_*) and interstitial pressure (*P*_*i*_). The difference *P*_*c*_ – *P*_*i*_ represents the hydraulic (water) pressure gradient across the capillary wall and is opposed by the osmotic pressure gradient (*π_*p*_* – *π_*i*_*) that retains fluid within the vessel.

Fluid filtration rate per unit area through microvascular walls (*J*_*V*_) can be written as (61):


(1) $$J_{V}=K\,\left[\left(P_{c}-P_{i}\right)-\sigma \,\left(\pi_{p}-\pi_{i}\right)\right]=K\,\left(\Delta \,P-\sigma \,\Delta \,\Pi \right)$$

where *K* is the hydraulic conductance (permeability) of microvascular vessel walls to fluid and is dependent on both their permeability coefficient and their surface area for filtration. σ is the Staverman’s reflection coefficient of the membrane for protein and accounts for the fact that the osmotic pressure difference across the permeable capillary wall is lower than the pressure difference across an impermeable membrane. The reflection coefficient has values between 0 and 1.0 (61). Macromolecules account for most of the colloid osmotic pressure (COP) gradient because the reflection coefficients of the small solutes are low (<0.1) resulting in a negligible concentration difference across microvascular walls.

A more modern view posits that in most vascular beds, there is a declining filtration along with the entire length of the capillaries, with an overall small net filtration force (62, 63). In this “revised” Starling model, the role of the endothelial glycocalyx layer (eGC) is emphasized as a key player in vascular permeability and fluid exchange. The eGC lies on the luminal endothelium and acts as a major barrier to the movement of molecules across the vascular wall, due to its tight entanglement and predominantly negatively charged structure that prevents albumin and other proteins transfer into the interstitial space (64).

**FIGURE 2:**
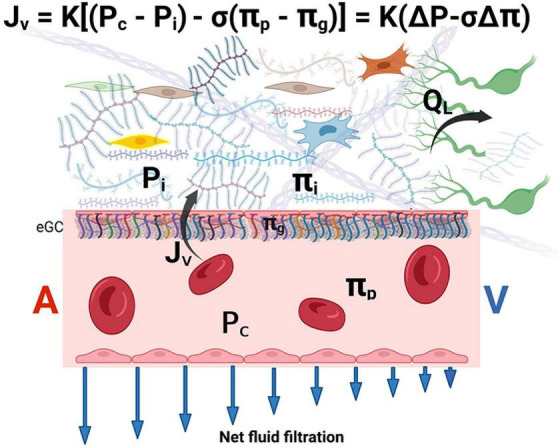
The revised Starling equation. Microvascular fluid exchange (flow *J*_*v*_) can be described by the revised Starling equation. The endothelial glycocalyx (eGC) layer modifies the colloid osmotic pressure that opposes filtration from being between the plasma and the interstitium to between *π_*p*_* and *π_*g*_* (where *π_*g*_* << *π_*i*_*). Blue arrows indicate that net force imbalance and flow diminish along with the length of the capillary from the arterial side (*A*) to the venous side (*V*) of the capillary, but minor filtration prevails even in venules. The interstitial pressure (*P*_*i*_) is the main determinant of lymph flow (*Q*_*L*_) in the initial lymphatics (see text for details). *K* is the hydraulic conductance (permeability) of microvascular vessel walls to fluid. σ is the Staverman’s reflection coefficient of the membrane for protein. *A* and *V* represent the arterial and venous sides of the capillary, respectively. eGC is the endothelial glycocalyx.

The eGC acts as an interface between blood and the capillary wall, which maintain a relatively low rate of filtration throughout the capillary length. The reflection coefficient of the eGC is high (σ ≥ 0.9) and its diffusional permeability to macromolecules is low. Plasma macromolecules largely cross the microvascular walls into the interstitium *via* openings in the glycocalyx. The COP immediately beneath the sub-eGC space (*π_*g*_*) is *lower* than that of the ISF (*π_*i*_*) since the space between the eGC and the endothelial cell membrane is nearly devoid of macromolecules. Therefore, osmotic pressure of the ISF does not directly determine transendothelial oncotic pressure difference that opposes filtration (65, 66). It follows that the eGC layer modifies the COP gradient from being between the plasma and the interstitium (*π_*p*_* – *π_*i*_*) to between the plasma and a nearly protein-free space between the eGC and the endothelial cell membrane (*π_*p*_* – *π_*g*_*) (62, 65–69). The later difference generates the COP that opposes filtration into the interstitial compartment and establishes a low *J*_*v*_ and lymph flow in most tissues (65, 66) (Figure 2).

Of note, *π_*g*_* cannot be measured directly but is supported by theoretical considerations and the experimental findings of “osmotic asymmetry” across continuous endothelium, namely, altering *π_*i*_* changes the filtration rate by only a small fraction of that predicted by the Starling principle (62, 65, 67). The concept of *π_*g*_* that operates just below the eGC also solves the inconsistency between the observed tissue lymph production that is much lower than the expected net capillary filtration rate calculated based on the larger *π_*i*_* (the “low lymph flow paradox”) (62, 67, 70).

According to this paradigm, at a steady state of normal hydrostatic pressures, no reabsorption from the interstitium into the intravascular space occurs in non-fenestrated capillaries (66). This leads to a slight net outwardly directed forces and flows into the interstitium (rather than true equilibrium), particularly at the early capillary portion. At the venular end of the capillary, a small driving force for fluid filtration remains, thus, establishing a no-reabsorption state throughout the capillary (Figure 2). Fluid leakage into the interstitium is physiologically responsible for tissue hydration and nutrition (71).

If the *P*_*c*_ transiently declines, fluid is reabsorbed from the interstitial compartment in capillaries and post-capillary venules only for a short duration (minutes to hours) (68). The reason for non-sustained microvascular reabsorption is that the ultrafiltration of the absorbed ISF at the underside of the eGC (of the blood capillary wall) raises subglycocalyx plasma protein concentration (*π_*g*_* and *π_*i*_*), and simultaneously, the *P*_*i*_ falls as fluid is removed from the interstitium. The changes in *π_*g*_* and *P*_*i*_ gradually abolish the net absorptive force so that, ultimately, a steady state of slight filtration is restored (65).

The steady state is maintained by low levels of filtration in most tissues. The interstitial COP changes dynamically and is inversely related to the capillary filtration rate. Raising the capillary filtration rate “dilutes” the macromolecules in the ISF (65). The COP differences are maintained in a steady state by a low filtration rate. Because water and small solutes are carried into the ISF faster than large protein molecules, protein concentration in the interstitium is considerably lower than that of plasma. Consequently, the COP difference between plasma and ISF can be maintained constant by the greater transcapillary flow rates of the fluid than protein (68). Note that given the inverse dependence between *π_*i*_* and *J*_*v*_, steady-state (but not transient) absorption in venous capillaries and venules is unlikely because the diminishing of *P*_*c*_ and *J*_*v*_ down the capillary results in increased *π_*i*_* (65).

Given an intact eGC (e.g., no inflammatory condition), the capillary hydrostatic pressure is the major determinant of fluid filtration. In HF, edema occurs when the high venous pressures produce high microvascular filtration that exceeds lymph drainage capacity. This implies a crucial role of lymphatic function in drainage of capillary filtrate and maintaining ISF balance when capillary pressure increases (65). The lymph flow is a part of the new steady state that builds with increased *P*_*c*_ and increases to match net capillary fluxes.

For example, experimental evidence suggests that the net pressure gradient favoring filtration must increase by ≥12–15 mmHg to produce edema (50, 59). Gradient tissue volume is expanded only slightly with smaller elevations of the filtration because of the increased lymph flow, the increase in *P*_*i*_, and the reduction in *π_*i*_*.

Disruption of glycocalyx integrity by various pathological conditions, such as sepsis, hyperglycemia, and hypotension, increases hydraulic conductivity resulting in increased vascular permeability and interstitial edema (72). Several studies suggest that natriuretic peptides (and therefore HF and volume overload) increase vascular permeability (73) by activating receptors on the microvascular endothelium (74), which causes shedding of the endothelial glycocalyx (75–77). In the pulmonary capillaries, extreme elevation of Pc can damage the eGC and cause stress failure leading to hemoptysis and edema (78).

## Transcapillary plasma refill

Effective decongestion with diuretics reduces PV, and consequently, capillary hydrostatic pressure (*P*_*c*_) is reduced. This causes ISF movement across the capillary into the intravascular compartment (plasma transcapillary refill) (79). The plasma refill rate (PRR) becomes important during common therapeutic interventions that reduce PV, such as diuretic therapy and ultrafiltration (79, 80). Fluid transport between the interstitial and intravascular compartments at the capillary level is governed not only by the hydrostatic and oncotic forces in both compartments (i.e., the Starling principle) but also by permeability and whole-body area of the capillary membranes. The average maximal PRR ranges from 5 to 6 ml/kg/h with substantial patient-to-patient variability in varying clinical circumstance (81).

Inadequate plasma refill is frequently evident during dialysis, as fluid is rapidly removed from the blood compartment by ultrafiltration. Plasma refill during dialysis is generally smaller than the ultrafiltration rate (82), and only partially compensates for the reduction in PV (83), hence, commonly leads to intradialytic hypotension (84). Large total volume removal during dialysis (particularly > 10 to > 13 ml/h/kg in different studies) is associated with an increased risk of intradialytic hypotension (81, 85). Fluid removal is better tolerated at rates of 150–250 ml/h (86) with ultrafiltration in HF patients. Patients with HF with preserved ejection fraction or predominant right-sided failure are particularly susceptible to intravascular volume depletion and generally require lower ultrafiltration rates (50–100 ml/h) (87). This may also explain, in part, the higher rates of worsening renal function observed during ultrafiltration in congested HF patients (37, 88).

However, the hemodynamic response to a rapid reduction in intravascular volume is complex and also depends on the efficacy of compensatory mechanisms, such as autonomic function, left ventricular function, and shunting blood from splanchnic and cutaneous beds into the central circulation, due to vasoconstriction (84, 85, 89, 90). Importantly, HF patients tend to develop intradialytic hypotension with lower ultrafiltration rates (91).

In acute HF patients undergoing decongestive therapy with diuretics, plasma refill mismatch may be a more subtle phenomenon because intravascular volume is reduced at a slower rate. However, insufficient volume replenishment (transcapillary refill and lymphatic refill) with volume contraction can activate baroreceptors in the aortic arch and carotid sinuses, leading to sympathetic nervous system activation with decrease in blood flow to the kidneys, decrease in glomerular filtration, increase in renin release, and increase renal sodium and water reabsorption (79, 92, 93). Errors in the assessment of the degree of volume overload or attempting to achieve dry weight that is too low set the stage for the occurrence of intravascular volume depletion. Therefore, continuation of aggressive diuresis in the face of diminished interstitial congestion may predispose to intravascular underfilling. However, this condition is only suspected or recognized after a significant reduction in urine output, pre-renal azotemia, or worsening of renal function has already occurred.

The true incidence of clinically relevant insufficient plasma refill is not known. The average maximal PRR of 5–6 ml/kg/h (∼350 ml/h for a 70 kg body weight) is infrequently achieved with aggressive combination diuretic therapy. PRR of the intravascular compartment from the congested interstitium is thought to decrease during decongestive therapy owing to the reduction in the interstitial hydrostatic pressure during therapy (79, 93). In heart failure, PRR may also be impaired due to cachexia with loss of plasma proteins and reduced plasma COP (2). In addition, *P*_*c*_ can remain elevated after diuresis secondary to persistent elevation of systemic venous pressure with right ventricular dysfunction (94, 95). Furthermore, PV measurements have shown that the intravascular compartment commonly remains expanded during diuretic therapy (43).

Inadequate PRR may imply that the patient remains with clinical evidence of congestion owing to the expanded interstitial compartment, while the intravascular volume is normal or low (88). The optimal clinical management of this condition remains unclear but concerns regarding this condition frequently lead to withdrawal of diuretics, neurohormonal blockers and/or fluids administration (96) with potential exacerbation of congestion.

The plasma refill rate cannot be readily measured, and no clinically validated tool is available to diagnose this condition. Hemoconcentration (i.e., an increase in hemoglobin/hematocrit in response to diuretic therapy) has been suggested as a putative metric to help guide inpatient decongestion (79, 97, 98). Because hemoconcentration is highly dependent upon the rate of fluid shifts (99, 100), it can also be observed during the generation of edema due to fluid loss from the intravascular compartment (101, 102). The lack of hemoconcentration is entirely consistent with efficient decongestion or with ineffective decongestion (both with matching refill). A rate of plasma removal exceeding the refill rate will not only temporarily lead to hemoconcentration when the desired complete interstitial decongestion is achieved but also at a time when there is still excess ISF.

Medical devices to monitor change and PRR during dialysis based on non-invasively monitors hematocrits by optical transmission have been advocated to better manage intradialytic volume removal (e.g., Crit-Line). However, in a randomized controlled trial, intradialytic blood volume monitoring was associated with higher non-vascular and vascular access–related hospitalizations and mortality (103).

Other tools, such as bioimpedance spectroscopy and lung ultrasound, can be used to assess changes in extracellular volume excess. Future technologies include nuclear magnetic resonance (NMR) relaxometry, providing a direct, non-invasive measurement of fluid volume, and interstitial pressure sensors (104). Portable, non-imaging, single-sided NMR sensor can rapidly assess clinically relevant changes in the fluid status of hypervolemic patients (105).

## The lymphatic system

The lymphatic system received little attention in the context of heart failure until recently (106–108). The lymphatic system is a blind-ended network of vessels that play an important role in mediating interstitial tissue fluid homeostasis by absorbing extravasated fluid and transporting it back to the venous circulation. Lymph flow also serves to remove proteins from the interstitial pool, thus lowering the interstitial oncotic pressure (60). In a normal steady state, the net transcapillary fluid flux into the interstitial compartment equals lymph flow and maintains a constant interstitial volume (50). Hence, ISF balance and homeostasis depend critically on adequate lymphatic function in most tissues (65). Because of ISF accumulates when the rate of transudation from capillaries into the interstitium exceeds the rate at which the lymphatic system can efficiently drain the fluid, edema can also be perceived as a failure of lymph drainage (63).

The initial lymphatics (the smallest lymphatic vessels) are composed of a single layer of endothelial cells with discontinuous basement membrane and anchoring filaments that project into the interstitium. The lymphatic endothelial cells lack tight cell–cell junctions and a continuous basement membrane (71). Rather, these cells form overlapping or interdigitating flaps with discontinuous button-like junctions (71, 109). Therefore, lymphatic capillaries are permeable and facilitate fluid absorption from the interstitium (110).

**FIGURE 3:**
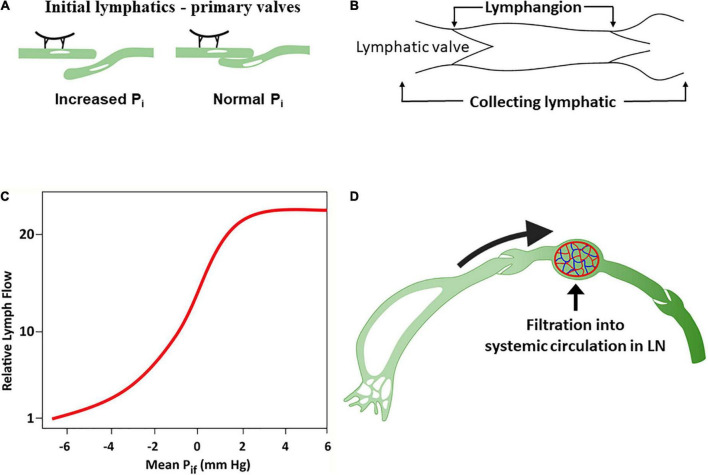
The lymphatic system. **(A)** Response of lymphatic capillaries to an increase in interstitial fluid volume. As the tissue matrix expands, tension on the anchoring filament rises, and the lymph capillaries open to allow entry of interstitial fluid into the initial lymphatics. **(B)** A lymphangion is defined as the segment between two unidirectional valves and typically exhibits systole and diastole. **(C)** Relationship between interstitial fluid pressure and lymph flow **(D)**. Under normal conditions, a substantial amount of lymph is reabsorbed in the nodal blood circulation depending on hydrostatic and osmotic forces acting at the blood–lymph barrier. Modified from references (58, 71).

The lymphatic endothelium is attached to the adventitial tissue by anchoring fibrillin filaments that connect the lymphatic endothelial cells with interstitial collagen and elastin fibers (111, 112). The tethering of initial lymphatics on their abluminal surfaces to adjacent tissues creates radial tension with openings between initial lymphatic endothelial cells when interstitial pressure rises (called “primary valves”) (111, 113, 114), acting as flap valves that allow unrestricted fluid transfer from the interstitium to the lumen (Figure 3A). Interstitial fluid accumulation serves to increase ISF pressure (*P*_*i*_) and forces entry into the initial lymphatics when *P*_*i*_ exceeds the initial lymphatics luminal pressure (115).

The initial lymphatic vessel segments coalesce into larger, collecting lymphatic vessels characterized by the presence of functional contractile units known as lymphangions – a vessel segment flanked by two valves (116) (Figure 3B). Lymphangions are encircled by smooth muscle cells that generate rhythmic synchronized contractions to the segments bounded by luminal bileaflet one-way valves (secondary valves), aimed to prevent fluid backflow (110). The lymphangions are organized in series and are capable of spontaneous pumping activity that propels lymph in a unidirectional manner, generating higher lymph pressures than individual lymphangions. Normal lymphatic pump function is determined by the intrinsic properties of lymphatic muscle, and the regulation of pumping is mediated by lymphatic preload and afterload, whereby the extent of contraction increases with the degree of lymphangion filling (71, 110, 117). Pumping also occurs through extrinsic means, such as adjacent tissue movement and muscle contractions, that compress and evacuate lymph vessels (71).

ISF pressure is the main determinant of uptake of ISF into initial lymphatics (50, 55). ISF accumulation increases the *P*_*i*_ surrounding the initial lymphatics. Initially, lymph flow increases linearly with the increased *P*_*i*_ in the interstitial low-compliance range (118) compensating for excess capillary filtration. However, with additional fluid transudation into the interstitium, the interstitial compliance increases, and *P*_*i*_ raises only modestly, resulting in relative reduction and subsequently a plateau in lymphatic flow which now fails to compensate for the excess capillary filtration (50, 119–121) (Figure 3C).

In humans, it is estimated that ∼8 L of fluid per day enters the interstitium and becomes afferent lymph. Generally, lymph is delivered by collecting lymphatics to at least one lymph node prior to entry into the thoracic duct (122). Transport of fluid through the blood–lymph barrier occurs within lymph nodes depending on the acting hydrostatic and osmotic pressures, resulting in fluid being transferred from the prenodal lymph into blood (Figure 3D). Lymph proteins can become concentrated to a variable degree during transit in lymph nodes (123, 124).

It is estimated that about half the fluid content of afferent lymph can be absorbed by lymph node microvessels (65, 125). After reabsorption to the bloodstream in lymph nodes capillaries and venules, the total postnodal (efferent) flow rate is estimated to be ∼4 L/day (125). The postnodal collecting lymphatics coalesce into larger lymphatic trunks, returning to the venous circulation (71, 126). The thoracic duct collects most of the lymph in the body other than from the right thorax, arm, head, and neck, which are drained by the right lymphatic duct (115).

In normal circulatory conditions of the arterial and venous systems, the ISF volume balance is maintained, and the lymph fluid is cleared back into the intravascular compartment. Adequate lymphatic vessel function becomes particularly important when excessive microvascular filtration occurs because of increased capillary pressure, such as HF. The thoracic duct lymph flow is significantly increased under such conditions, as demonstrated in the experimental right (127) and left (128) HF and in patients with HF (129, 130). This is also well-documented in the lung, where the lymphatic vasculature markedly increases its drainage capacity in response to chronic elevation of left atrial (LA) pressure (131, 132). This functional expansion of the pulmonary lymphatic system has also been demonstrated in patients with mitral stenosis, where dilated lymphatics in the pleura and interlobular septa were observed, and in some cases, the lymphatics had acquired a hypertrophied muscular wall (133).

Overall, the lymphatic system can increase fluid removal by at least >10-fold higher (and up to 50-fold) than the normal amount of fluid entering the interstitium in response to increased hydrostatic pressure (60, 120, 130, 131), and this provides a large “safety factor” against edema formation. The augmentation of lymph flow is critically dependent on the changes in interstitial pressure (60) (Figure 3C). Therefore, clinical edema formation requires a substantial increase in the pressure gradients favoring filtration (59). Importantly, however, in the setting of heart failure with fluid retention and peripheral edema, adequate lymphatic function in isolation can only restore fluids from the expanded interstitium into a vascular compartment, where PV is already increased.

Eventually, the lymphatic vessels of patients with HF exhibit structural and molecular alterations and cannot effectively compensate for fluid extravasation and interstitial accumulation (107). Lymphatic pump failure has been described in human breast cancer–related lymphoedema (71). Similarly (albeit with fewer supporting data), chronic HF may lead to progressive dysfunction of the lymphatic system akin to lymphedema, namely, weakening of the active lymphatic contractions, chronic distension of the collecting vessels, and consequent incompetence of the lymphatic valves (106, 134). In patients with severe HF and long-standing edema, the superficial lymphatics are widely distended with extravasation back into tissues. These vessels may fail to demonstrate forward flow or show retrograde flow secondary to extreme dilatation of the lymphatics that renders the valves dysfunctional (134). The thoracic duct and its tributaries can be markedly dilated, with ballooning of the duct at the junction with the jugular vein (130).

### Role of the central venous pressure

Lymph flow in the thoracic duct is dependent on the intrathoracic pressure, the pressure generated by the duct contractile elements, and the venous backpressure in the subclavian vein (71, 135). The pressure gradient between the thoracic duct and the lymphatic duct is reduced as the central venous pressure typically increases to level as high as ∼15 mmHg at the outflow of the ducts (127, 136, 137) analogous to an elevation in afterload on the lymphatic duct. This impairs the ability of a lymphatic system to clear the additional fluid that accumulates in the peripheral interstitial compartment and air spaces of the lungs.

It has been demonstrated in animal studies that increased central venous pressure at the site of the thoracic duct outlet impacts lymphatic drainage (138). By contrast, the thoracic duct lymph flow was enhanced when the thoracic duct was positioned *via* a shunt diverting the flow around the failing right heart into the low-pressure pulmonary veins (127), and when the cervical portion of the thoracic duct was cannulated and removed by gravity drainage (130). This finding is surprising given that the lymphatic contraction can generate pressures ranging from 20 to 120 mmHg in the recumbent and upright positions, respectively (106), suggesting that the lymphatic contractile function may be impaired in chronic HF.

**FIGURE 4:**
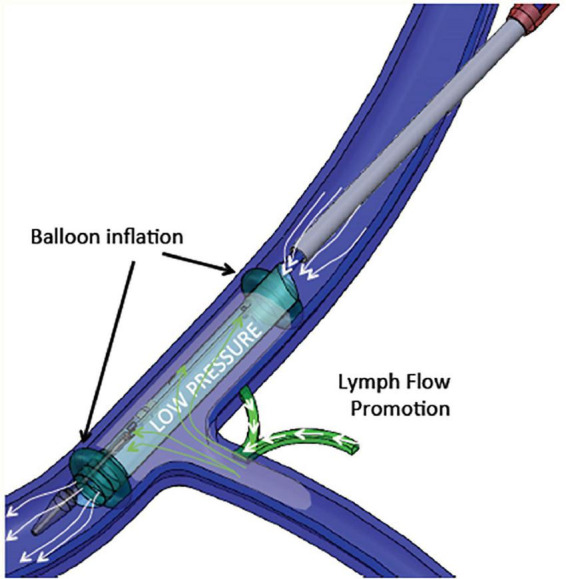
WhiteSwell System mechanism of action. The system promotes fluid movement from the thoracic duct into the internal jugular vein. Polyurethane balloons mounted on the catheter are inflated to isolate the subclavian vein from the internal jugular vein. As the balloons are inflated, an inner membrane is expanded, providing a lumen for blood flow through the jugular vein between the inflated balloons. Suction is applied to the isolated area between the two inflated balloons, which creates a low-pressure zone (2–5 mmHg) between the inflated balloons.

Therefore, increased central venous pressure may provide progressive resistance to the return of lymph to the venous circulation and reduces lymph flow in the thoracic duct (136, 139). A device-based approach (WhiteSwell, Israel) was designed to create a low-pressure zone (2–5 mmHg) in the outflow area of the thoracic duct (Figure 4). This low-pressure zone promotes the physiological process of lymph flow from the interstitial space into the intravascular compartment. By promoting a fluid shift from the congested interstitium to the intravascular space, the device may facilitate the effect of a diuretic regimen, while avoiding hypotension and worsening of renal function that can occur due to diuresis-induced intravascular volume depletion. In a sheep model of induced HF and acute volume overload, the device decreased extravascular lung water compared with controls (108).

## The starling principle in the context of congestion and decongestion

**FIGURE 5:**
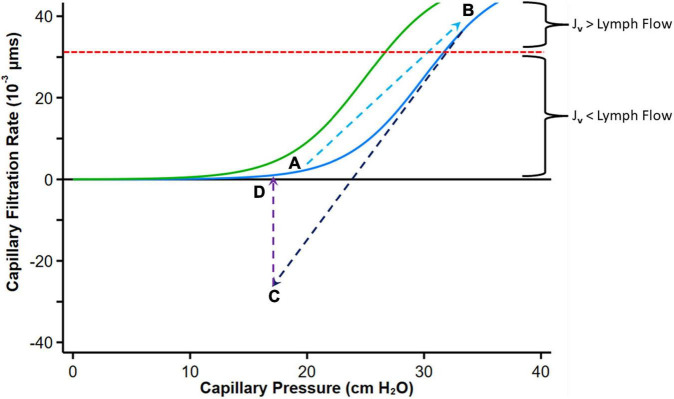
Graph depicting fluid exchange (filtration or absorption rate, *J*_*v*_) across the capillary wall as a function of capillary pressure. Point A represents a normal steady state position with minimal filtration. With heart failure decompensation, fluid and salt retention cause an increase in mean capillary pressure, leading to an increase in fluid absorption into the interstitial compartment and a reduction in plasma COP which shifts the curve to the left (Green curve, Point B). Diuretic therapy reduces capillary pressure with reversal to fluid absorption into the intravascular compartment (Point C). If diuresis is brief, the absorption rate falls and can move again toward Point B. Fluid uptake into the intravascular compartment has a variable effect on plasma protein concentration depending on the relative rates of diuresis and plasma refill rates. When diuresis increases the interstitial COP, the colloid osmotic pressure difference declines and reduces absorption of fluid into the capillaries (Point D). The red dashed line indicates the point where the maximal removal capacity of lymph flow cannot compensate for the increased capillary filtration. Modified from references (61, 65, 66). See text for details.

The curve describing the relationship between hydrostatic pressure gradient and filtration rate into the interstitial space has been called “hockey stick” or “J” curve under steady state conditions (61, 66, 68, 69) (Figure 5). The sharp change (inflection) from low slope to steep slope generally occurs just below the COP of plasma. At low *P*_*c*_ transendothelial filtration is minimal (Figure 5, Point A) but above the inflection point, the COP difference opposing filtration is maximal, and *J*_*v*_ is essentially proportional to Δ*P* which is mainly determined by *P*_*c*_ (66).

With volume expansion in heart failure (as during a period of decompensation), the *P*_*c*_ gradually increases and *π_*p*_* may decline, both favoring filtration into the interstitium akin to the infusion of solution with no colloid osmotic pressure. Fluid is retained within the intravascular compartment until Δ*P* increases above the inflection point. Filtration into the interstitial compartment results in protein dilution (59, 65), leading to a progressive fall in *π_*i*_* (or more accurately *π_*g*_*), and both the resorptive force *π_*p*_* – *π_*g*_* (with a left shift of the curve) and *P*_*i*_ increase until a new steady state is reached (Figure 5, Point B).

If flow into the interstitium exceeds the compensatory lymphatic reserve tissue edema occurs. Thus, in contrast to the normal “no absorption” steady state in capillaries and venules where filtered fluid is returned to the circulation mostly by lymphatics, the presence of interstitial edema implies that the lymphatic reserve is exhausted. Consequently, it can be postulated that during decongestive therapy with diuretic or ultrafiltration, lymphatic flow can only marginally increase (with the reduction in central filling pressures), and fluid largely returns to the intravascular compartment *via* capillary absorption.

Diuretics enhance the loss of sodium and fluids *via* the kidneys, leading to a reduction in PV and central filling pressures with a reduction in *P*_*c*_ (and Δ*P*) with fluid reabsorption into the intravascular compartment (Figure 5, Point C). A concomitant augmentation of *π_*p*_* may occur owing to the concentration of plasma proteins if diuresis exceed PRR (140), which also facilitates ISF reabsorption into the intravascular compartment. According to this paradigm, absorption can revert to filtration as proteins from the ISF diffuse into the subglycocalyx space increasing *π_*g*_* (66); the overall COP pressure difference falls, with the resulting reduction of absorption rate into the intravascular space and eventually cessation of transcapillary plasma refill and establishment of a new steady state (Figure 5D).

Thus, with any interruption to plasma volume reduction by diuresis that only leads to a transient reduction in *P*_*c*_, this process will not cause sustained absorption of ISF (complete decongestion), and the sum of forces is expected to move again toward filtration. However, at this point, if *P*_*c*_ remains lower than its initial value, filtration into the interstitium is reduced and the lymphatic flow may now be sufficient to prevent progressive edema.

A more effective interstitial decongestion, therefore, requires a substantial reduction in PV (and *P*_*c*_) that is prolonged and sustained (or several step reductions in *P*_*c*_), which is not always achieved in clinical practice. Sustained efficient diuresis can increase *π_*p*_* if the plasma refill rate does not match diuresis (this may also occur if the interstitium is no longer congested). The rise in *π_*p*_* shifts the curve with its inflection region to the right (its original position), thus requiring higher Δ*P* to produce edema.

Finally, although Δ*P* increases in HF to favor fluid filtration into the tissue, low arterial blood pressure, autonomic reflexes, and medications can affect Δ*P* by altering pre-capillary and post-capillary resistances of the microcirculation. Vasodilators that dilate arterioles increase the hydrostatic pressure within the capillary bed (90). Acute reduction in Δ*P* by pre-capillary vasoconstriction (70), post-capillary vasodilation, or hypovolaemia can result in absorption of fluid from the interstitium that is generally transient.

## Interstitial decongestion *via* the skin

Sweat glands are perceived to play an important excretory function, similar to that of the renal system, responsible for clearing excess micronutrients, metabolic waste, and toxicants from the body. However, the effectiveness of sweat glands as an excretory organ for homeostatic purposes remains unclear. Eccrine glands are the major sweat glands of the human body and are distributed across nearly the entire body surface area. These sweat glands are responsible for the greatest volume of sweat excretion (141, 142).

The eccrine gland opens out through the sweat pore and thus produces a clear, odorless fluid, consisting primarily of water and electrolytes. The precursor fluid that enters the secretory coil of the swat gland (primary sweat) is the ISF (142), and therefore, nearly isotonic with blood plasma with respect to electrolytes. Fluid flowing through the eccrine gland duct is passively reabsorbed *via* several ion channels, including the epithelial sodium channels (ENaCs) on the luminal membrane and actively reabsorbed *via* Na^+^/K^+^-ATPase transporters on the basolateral membrane. Chloride is passively reabsorbed *via* the cystic fibrosis transmembrane conductance regulator (CFTR) on the luminal and basolateral ductal cell membrane (143, 144). The final result is hypotonic sweat excreted onto the skin surface (143, 144). With increased sweat rate, Na^+^ and Cl^–^ secretion in precursor sweat increases proportionally more than the reabsorbed Na^+^ and Cl^–^, and therefore leads to higher final sweat Na^+^ and Cl^–^ concentrations (143–146).

Eccrine sweat glands primarily respond to thermal stimuli, predominantly an increase in body core temperature. However, skin temperature also independently affects sweat rate such that local heating accentuates sweat rate, while local cooling attenuates sweat rate (147). The perspiration threshold of skin temperature is ∼33 to 34°C, above which the sweating rate is linearly dependent on skin temperature (147, 148).

Humans can produce large amounts (>1 L/h) of sweat during prolonged exercise in the heat (149). Although sweat is hypotonic (with respect to Na^+^ and Cl^–^) (143), a substantial amount of sodium can be removed *via* sweat. Na^+^ concentration in sweat increases linearly with increases in sweat rate (146, 150) and is generally in the range of 20 and 80 mmol/L (151, 152). However, higher concentrations can be achieved, as the sodium and chloride concentrations increase with increasing sweat rate (145, 153).

A device proposed by AquaPass Medical (AquaPass Medical Ltd., Shefayim, Israel) makes the use of this natural pathway for ISF excretion to remove excess fluids directly from the interstitial compartment. Because the interstitial compartment is decongested directly, this method is independent of renal function, diuretic efficacy, and plasma refill.

**FIGURE 6:**
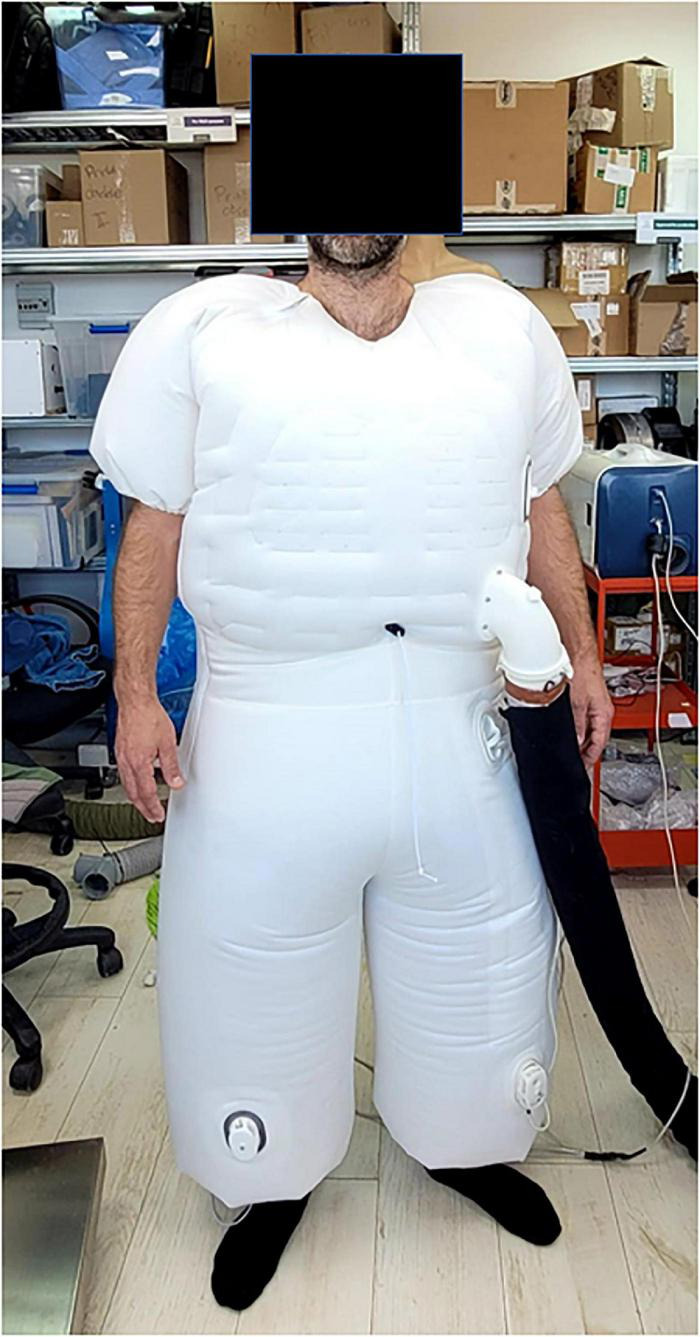
The AquaPass device.

The device is designed to accommodate the patient’s body from the foot and the entire torso (Figure 6). It creates a homogeneous warm temperature environment around the lower part of the body and torso leading to increased skin temperature that initiates perspiration. The system ensures, however, that the body core temperature remains within normal range. In addition, because patient’s comfort is paramount with this form of therapy, the sweat evaporates instantaneously, thus avoiding the awareness of perspiration by the patient and enabling long durations of treatments, if required.

The skin starts to increase sweat production at temperatures of 33°C and reaches optimal sweat rate at 38°C. Above 39–40°C, there is a risk of uncomfortable sensation and overheating (154). Therefore, the target of the AquaPass system is to create a uniform temperature surrounding, optimally between 36 and 38°C, where the slope of the relationship between temperature and sweat production is linear (148, 155, 156).

Based on initial results from an ongoing first-in-man study in healthy volunteers, the AquaPass device is able to remove fluids at a rate of 150–250 ml/h (157). This fluid removal rate is equivalent to that of effective diuretic therapy (2).

## Conclusion

Failure of complete decongestion is often attributed to diuretic resistance and renal dysfunction. Control of fluid overload can be achieved by the kidneys only if interstitial fluids readily equilibrate. Therefore, even in patients with relatively preserved renal function and a favorable response to diuretics, effective interstitial decongestion requires adequate lymphatic flow and plasma refill. The fundamental processes that underlie incomplete interstitial decongestion are often ignored. These include dysfunction of the lymphatic system, the interstitial pressure–volume curves, and the factors governing the movement of fluids between the interstitial and vascular compartments. The critical importance of the interstitium in the congestive state underscores the need to directly decongest the interstitial compartment without relying on the lowering of intracapillary pressure with diuretics. This critical unmet need may be addressed by novel device therapies in the near future.

## Author contributions

The author confirms being the sole contributor of this work and has approved it for publication.
